# Precision Joint RF Measurement of Inter-Satellite Range and Time Difference and Scalable Clock Synchronization for Multi-Microsatellite Formations

**DOI:** 10.3390/s23084109

**Published:** 2023-04-19

**Authors:** Cong Hou, Xiaojun Jin, Lishan Zhou, Haoze Wang, Xiaopeng Yang, Zhaobin Xu, Zhonghe Jin

**Affiliations:** 1 School of Aeronautics and Astronautics, Zhejiang University, Hangzhou 310027, China; 12024045@zju.edu.cn (C.H.); axemaster@zju.edu.cn (X.J.); 22124050@zju.edu.cn (L.Z.); 22124024@zju.edu.cn (H.W.); 22224060@zju.edu.cn (X.Y.); jinzh@zju.edu.cn (Z.J.); 2 Key Laboratory of Micro-Nano Satellite Research, Hangzhou 310027, China; 3 Micro-Satellite Research Center, Zhejiang University, Hangzhou 310027, China

**Keywords:** multi-satellite formations, time difference measurement, ADS-TWR, joint RF measurement, clock synchronization

## Abstract

The rapid development of multi-satellite formations requires inter-satellite radio frequency (RF) measurement to be both precise and scalable. The navigation estimation of multi-satellite formations using a unified time reference demands the simultaneous RF measurement of the inter-satellite range and time difference. However, high-precision inter-satellite RF ranging and time difference measurements are investigated separately in existing studies. Different from the conventional two-way ranging (TWR) method, which is limited by its reliance on a high-performance atomic clock and navigation ephemeris, asymmetric double-sided two-way ranging (ADS-TWR)-based inter-satellite measurement schemes can eliminate such reliance while ensuring measurement precision and scalability. However, ADS-TWR was originally proposed for ranging-only applications. In this study, by fully exploiting the time-division non-coherent measurement characteristic of ADS-TWR, a joint RF measurement method is proposed to obtain the inter-satellite range and time difference simultaneously. Moreover, a multi-satellite clock synchronization scheme is proposed based on the joint measurement method. The experimental results show that when inter-satellite ranges are hundreds of kilometers, the joint measurement system has a centimeter-level accuracy for ranging and a hundred-picosecond-level accuracy for time difference measurement, and the maximum clock synchronization error was only about 1 ns.

## 1. Introduction

As the importance of microsatellite formations has increased [1,2], high-precision inter-satellite ranging and clock synchronization, two inseparable key supporting technologies for the relative navigation of microsatellite formations, have received increasing attention. Inter-satellite clock synchronization can be established based on the inter-satellite time difference measurement, thereby providing a unified time reference for inter-satellite range measurement-based navigation solutions.

The inter-satellite range and time difference can be measured using the navigation satellite common view method or the inter-satellite RF autonomous measurement method [3,4]. The inter-satellite RF autonomous measurement method can effectively overcome the problems of the navigation satellite common view method, which has a high measurement accuracy only for low earth orbits and thus a limited scope of application. In addition, the inter-satellite RF autonomous measurement method does not rely on any external system and has a high degree of autonomy. Hence, it has been applied widely. Typical examples of application include PRISMA (Prototype Research Instruments and Space Mission Technology Advancement), GRACE (Gravity Recovery and Climate Experiment), GRAIL (Gravity Recovery and Interior Laboratory), and GRACE-FO (Gravity Recovery and Climate Experiment—Follow On) microsatellite formations. The PRISMA formation carried a two-way ranging (TWR)-based S-band inter-satellite range and angle measurement system [5], while the GRACE, GRAIL, and GRACE-FO formations carried a TWR-based K-band ranging system [6,7,8,9]. In addition, the GRAIL formation was equipped with an S-band time transfer assembly to realize inter-satellite time difference measurement and clock synchronization in a lunar orbit.

Although the existing RF measurement research has gained some achievements, two prominent problems still remain. One is that the high-precision inter-satellite RF ranging and time difference measurement have only been investigated separately in existing studies [10,11]. This means that a satellite needs to be equipped with two separate devices, one for inter-satellite ranging and the other for inter-satellite time difference measurement (for example, the GRAIL formation), resulting in an excessively complex space-borne navigation system. Some researchers [12,13] proposed a joint range and time difference measurement model for ground wireless sensor networks. However, there are two major deficiencies with these studies. First, the model was proposed for application scenarios with very low dynamics and had only a sub-meter measurement accuracy. Second, these studies focused on the localization estimation algorithm and did not consider the sources of measurement errors and provided no means to implement the joint measurement. Obviously, there is still a long way to go for the realization and application of space-borne high-precision joint measurement.

The other problem of the existing RF measurement research resides in the lack of a precision RF measurement method for miniaturized space-borne navigation systems. The traditional time division multiple access (TDMA)-based TWR method can effectively overcome the disadvantage of frequency division multiple access (FDMA) and code division multiple access (CDMA) based methods in terms of poor scalability and has been successfully applied to the inter-satellite links of the global positioning system (GPS) and Beidou navigation constellations [14]. However, the high measurement accuracy achieved with TWR in these navigation constellations is heavily reliant on high-performance atomic clocks and the assistance of navigation ephemeris, which are not available on microsatellite platforms due to limited resources.

In a previous work [15], authors proposed an ADS-TWR-based multi-satellite inter-satellite ranging scheme, and high-ranging accuracy could be achieved with only a common miniaturized frequency source and without any external assistance. However, ADS-TWR was originally proposed for ranging-only applications, so a separate time difference measurement equipment is still needed to constitute a space-borne navigation system [16,17]. To this end, by fully exploiting the non-coherent measurement characteristic of ADS-TWR that potentially supports joint measurement, this study proposes a multi-satellite joint inter-satellite range and time difference measurement method.

It is noteworthy that inter-satellite time difference cannot be obtained directly as an inter-satellite range. Therefore, a critical problem of establishing a higher precision time difference reference needs to be solved to validate the time difference measurement performance. To address this issue, a CDMA-based high-precision time difference reference design approach is proposed to evaluate the time difference measurement performance of the joint measurement method. This reference is implemented on the same hardware platform of the joint measurement system and thus has the advantages of simplicity and miniaturization.

Regarding the clock synchronization method, the network time protocol (NTP) structure is generally used to achieve time synchronization for terrestrial wireless sensor networks. It works following three steps. First, part of the sensors in the network synchronizes 34 with the root node which possesses the reference time. Then, these sensors become new reference nodes. This process is passed layer by layer until all nodes in the network complete time synchronization [18]. However, this scheme has a problem in that errors will accumulate layer by layer and the synchronization accuracy depends heavily on the time accuracy of the root node. The consensus network [19] collects the clock information of all nodes, calculates the virtual clock of the current formation, and then each node is synchronized to the virtual clock. The consensus network scheme effectively solves the problems of NTP. However, the synchronization moment of all nodes must be strictly consistent, otherwise, the process will fail. From this point of view, this scheme is not suitable for TDMA. This is because, in a TDMA system, satellites can only communicate with other satellites in their own time slots for clock synchronization and cannot synchronize all the time like FDMA and CDMA. Meanwhile, in a consensus network, the clock failure of any node in the network will cause all nodes to lose synchronization with the virtual clock.

Based on the work of the joint measurement method, this paper further proposed an inter-satellite clock synchronization scheme for multi-satellite formations. This scheme can effectively alleviate the influence of frequency deviation among different satellites in a formation. It has the advantages that the synchronization error is not accumulated, its synchronization accuracy is not limited to a single node, and the clock synchronization is minimally affected by the failure of one satellite’s clock.

The joint measurement and clock synchronization methods proposed in this paper enable large-scale formation microsatellites to achieve precision inter-satellite range and time difference measurement and clock synchronization with only one set of equipment.

## 2. System Model

The researchers [15,20] described a TDMA and ADS-TWR-based method for distributed multi-satellite measurement. Because the ADS-TWR method was originally proposed for ranging applications, they only analyzed and simulated the inter-satellite ranging performance. In this study, the feasibility of the method for inter-satellite time difference measurement will be analyzed. On this basis, a model for high-precision joint measurement will be established.

**Figure 1:**
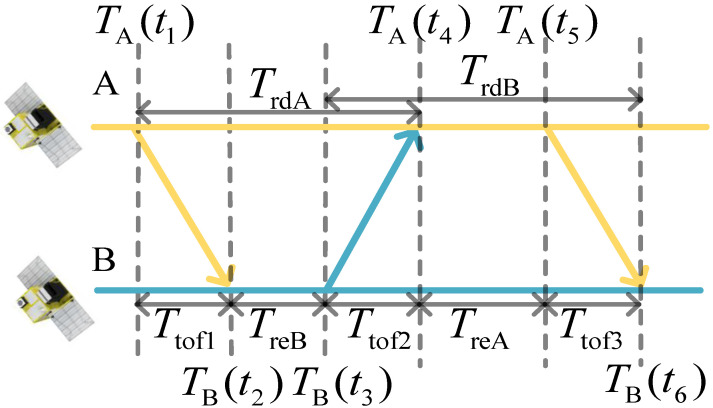
Principle of asymmetric double-sided two-way ranging.

An ADS-TWR measurement involves three signals between any two satellites in a formation, as shown in Figure 1. Through pseudo-noise (PN) code measurement, satellite B can obtain the time messages of $T_{A}{(t}_{1})$, $T_{B}{(t}_{2})$, $T_{A}{(t}_{5}),$ and $T_{B}{(t}_{6})$. Similarly, satellite A can obtain $T_{B}{(t}_{3})$ and $T_{A}{(t}_{4})$. $T_{\mathrm{tof}1}$, $T_{\mathrm{tof}2}$, and $T_{\mathrm{tof}3}$ are the times of flight.

According to the above time messages, satellite B can construct the time intervals between the signal transmission and reception, namely $T_{\mathrm{rdA}}$, $T_{\mathrm{rdB}}$, $T_{\mathrm{reA}},$ and $T_{\mathrm{reB}}$ as follows:

(1) $$\begin{array}{l} T_{\mathrm{rdA}}=T_{A}(t_{4})-T_{A}(t_{1}),T_{\mathrm{rdB}}=T_{B}(t_{6})-T_{B}(t_{3})\\ T_{\mathrm{reA}}=T_{A}(t_{5})-T_{A}(t_{4}),T_{\mathrm{reB}}=T_{B}(t_{3})-T_{B}(t_{2}) \end{array}$$

The subscript rd represents the round-trip time and re represents the reply time.

Based on the ADS-TWR time measurement, the distance, *R*, between the two nodes can be obtained as follows [20]:

(2) $$R=\frac{c}{4}\cdot \left[\left(T_{\mathrm{rdA}}-T_{\mathrm{reB}}\cdot \frac{T_{\mathrm{rdA}}+T_{\mathrm{reA}}}{T_{\mathrm{rdB}}+T_{\mathrm{reB}}}\right)\right.\left.+\left(T_{\mathrm{rdB}}-T_{\mathrm{reA}}\cdot \frac{T_{\mathrm{rdB}}+T_{\mathrm{reB}}}{T_{\mathrm{rdA}}+T_{\mathrm{reA}}}\right)\right]$$

where *c* is the light speed. Based on the range measurement and the times at the two nodes, the time difference, Δ*T*, between the two nodes at time $t_{3}$ can be obtained as follows:

(3) $$\Delta T=T_{A}\left(t_{4}\right)-R/c-T_{B}\left(t_{3}\right)=\left[T_{A}\left(t_{3}\right)-T_{B}\left(t_{3}\right)\right]+\left[T_{A}\left(t_{4}\right)-T_{A}\left(t_{3}\right)-R/c\right]$$

Equations (2) and (3) constitute the basic model for joint measurement.

The high-precision joint measurement of inter-satellite range and time difference in multi-satellite formations requires in-depth analysis and modeling of the sources of measurement errors, and particular attention needs to be paid to the difference between the time difference measurement errors and ranging errors. The major sources of measurement errors of the multi-satellite joint measurement method proposed in this study include the frequency deviation of the frequency source and satellite motion-induced dynamics. In addition, because the measurement system uses PN code measurement signals, PN code phase jitter noise, receiver thermal noise, and dynamic stress error are inevitable, which are hereafter referred to collectively as phase tracking noise [11,21]. Moreover, the ionosphere delay and hardware delay need to be corrected. The influence of each error source on ranging and the corresponding compensation measures can be found in [20]. For the time difference measurement, the influence of each error source and the compensation measures are similar to ranging.

Since the first and third signals in the ADS-TWR method are symmetric with respect to the second signal, the range between the two satellites at time $t_{3}$ is used as the reference range $R_{\mathrm{AB}}$, with the corresponding time of flight designated as $T_{\mathrm{tofAB}}$. Considering the above sources of errors, with the ionosphere delay and hardware delay corrected (the detailed correction is shown in Appendix A), the total ADS-TWR ranging error and time difference measurement error, $E_{R}$ and $E_{\Delta T}$, can be numerically modeled as follows:

(4) $$\begin{array}{ll} E_{R} & =R_{\mathrm{AB}}\cdot \left(\frac{K_{A}+K_{B}}{2}-1\right)+n_{R}+\frac{c}{4}\cdot \left(T_{\mathrm{tof}3}-T_{\mathrm{tof}1}\right)\cdot \frac{T_{\mathrm{reB}}\cdot K_{A}}{T_{\mathrm{tof}2}+T_{\mathrm{tof}3}+T_{\mathrm{reA}}+T_{\mathrm{reB}}}\\ & -\frac{c}{4}\cdot \left(T_{\mathrm{tof}3}-T_{\mathrm{tof}1}\right)\cdot \frac{T_{\mathrm{reA}}\cdot K_{B}}{T_{\mathrm{tof}1}+T_{\mathrm{tof}2}+T_{\mathrm{reB}}+T_{\mathrm{reA}}}+\frac{c}{4}\cdot \left[\begin{array}{l} \left(T_{\mathrm{tof}1}+T_{\mathrm{tof}2}-2T_{\mathrm{tofAB}}\right)\cdot K_{A}+\\ \left(T_{\mathrm{tof}2}+T_{\mathrm{tof}3}-2T_{\mathrm{tofAB}}\right)\cdot K_{B} \end{array}\right] \end{array}$$

(5) $$\begin{array}{l} E_{\Delta T}=T_{\mathrm{tof}2}\cdot K_{A}-\frac{E_{R}}{c}-T_{\mathrm{tofAB}}+n_{\Delta T}\\ =\frac{T_{\mathrm{tofAB}}\cdot (K_{A}-K_{B})}{2}+(T_{\mathrm{tof}2}-T_{\mathrm{tofAB}})\cdot K_{A}-\frac{1}{4}\cdot \left(T_{\mathrm{tof}3}-T_{\mathrm{tof}1}\right)\cdot \frac{T_{\mathrm{reB}}\cdot K_{A}}{T_{\mathrm{tof}2}+T_{\mathrm{tof}3}+T_{\mathrm{reA}}+T_{\mathrm{reB}}}\\ +\frac{1}{4}\cdot \left(T_{\mathrm{tof}3}-T_{\mathrm{tof}1}\right)\cdot \frac{T_{\mathrm{reA}}\cdot K_{B}}{T_{\mathrm{tof}1}+T_{\mathrm{tof}2}+T_{\mathrm{reB}}+T_{\mathrm{reA}}}-\frac{1}{4}\cdot \left[\begin{array}{l} \left(T_{\mathrm{tof}1}+T_{\mathrm{tof}2}-2T_{\mathrm{tofAB}}\right)\cdot K_{A}+\\ \left(T_{\mathrm{tof}2}+T_{\mathrm{tof}3}-2T_{\mathrm{tofAB}}\right)\cdot K_{B} \end{array}\right]\\ \;+n_{\Delta T}-n_{R} \end{array}$$

where $K_{A}$ and $K_{B}$ represent the ratios of the onboard clock frequency to the nominal frequency for satellites A and B, respectively, $n_{R}$ is the phase tracking noise error of ranging, and $n_{\Delta T}$ is the sum of the phase tracking noise errors at times $T_{A}{(t}_{4})$ and $T_{B}{(t}_{3})$.

The measurement parameters are listed in Table 1. These parameters are used in all experiments unless otherwise noted.

Although longer coherent integration time can improve the carrier-to-noise ratio of the received signal, it will also increase the frequency error. Furthermore, the coherent integration time must be less than the data bit period to avoid the influence of code polarity change. Thus, a 1/4 data bit period is selected as the coherent integration time, which is 50 μs.

### 2.1. Error Caused by Frequency Deviation

The dominant component of the error caused by frequency deviation in Equation (5) can be expressed as:

(6) $$C_{\Delta T}=T_{\mathrm{tofAB}}\cdot (K_{A}-1)-T_{\mathrm{tofAB}}\cdot [(K_{A}+K_{B})/2-1]=T_{\mathrm{tofAB}}\cdot (K_{A}+K_{B})/2$$

**Figure 2:**
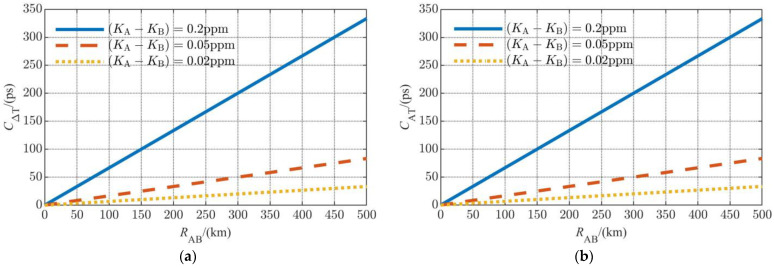
Numerical results of the error caused by frequency deviation: (**a**) time difference measurement error; (**b**) range measurement error (normalized to time).

The simulation result of the time difference measurement is shown in Figure 2a, where the ${(K}_{A}{+K}_{B})/2$ are 0.2 ppm, 0.05 ppm, and 0.02 ppm. For comparison, Figure 2b depicts the result of the range measurement, which is normalized to time by being divided by the speed of light.

Considering that the probability density functions of ${(K}_{A}{+K}_{B})/2-1$ and ${(K}_{A}-K_{B})/2$ are exactly the same, the influence of the frequency deviation on ranging and the time difference measurement is the same. For a typical inter-satellite distance of 200 km (the inter-satellite distance of the GRAIL formation is 175–225 km, and that of GRACE-FO is about 220 km) and a frequency accuracy of 0.01 ppm (which is available for a state-of-the-art miniaturized frequency source), the time difference measurement error caused by the frequency deviation can be controlled under 10 ps.

### 2.2. Motion Error

Due to the fast movement of LEO satellites, $T_{\mathrm{tof}1}$, $T_{\mathrm{tof}2}$, and $T_{\mathrm{tof}3}$ are not equal, which leads to the satellite motion error. The time difference measurement error caused by satellite motion in Equation (5) can be expressed as:

(7) $$\begin{array}{ll} M_{\Delta T} & =(T_{\mathrm{tof}2}-T_{\mathrm{tofAB}})\cdot K_{A}-\frac{1}{4}\cdot \left[\begin{array}{l} \left(T_{\mathrm{tof}1}+T_{\mathrm{tof}2}-2T_{\mathrm{tofAB}}\right)\cdot K_{A}+\\ \left(T_{\mathrm{tof}2}+T_{\mathrm{tof}3}-2T_{\mathrm{tofAB}}\right)\cdot K_{B} \end{array}\right]\\ & -\frac{1}{4}\cdot \left(T_{\mathrm{tof}3}-T_{\mathrm{tof}1}\right)\cdot \left(\begin{array}{l} \frac{T_{\mathrm{reB}}\cdot K_{A}}{T_{\mathrm{tof}2}+T_{\mathrm{tof}3}+T_{\mathrm{reA}}+T_{\mathrm{reB}}}-\\ \frac{T_{\mathrm{reA}}\cdot K_{B}}{T_{\mathrm{tof}1}+T_{\mathrm{tof}2}+T_{\mathrm{reB}}+T_{\mathrm{reA}}} \end{array}\right) \end{array}$$

Correspondingly, the range measurement error caused by satellite motion in Equation (4) can be expressed as:

(8) $$\begin{array}{ll} M_{\mathrm{AT}} & =\frac{c}{4}\cdot \left[\begin{array}{l} \left(T_{\mathrm{tof}1}+T_{\mathrm{tof}2}-2T_{\mathrm{tofAB}}\right)\cdot K_{A}+\\ \left(T_{\mathrm{tof}2}+T_{\mathrm{tof}3}-2T_{\mathrm{tofAB}}\right)\cdot K_{B} \end{array}\right]\\ & +\frac{c}{4}\cdot \left(T_{\mathrm{tof}3}-T_{\mathrm{tof}1}\right)\cdot \left(\begin{array}{l} \frac{T_{\mathrm{reB}}\cdot K_{A}}{T_{\mathrm{tof}2}+T_{\mathrm{tof}3}+T_{\mathrm{reA}}+T_{\mathrm{reB}}}-\\ \frac{T_{\mathrm{reA}}\cdot K_{B}}{T_{\mathrm{tof}1}+T_{\mathrm{tof}2}+T_{\mathrm{reB}}+T_{\mathrm{reA}}} \end{array}\right) \end{array}$$

**Figure 3:**
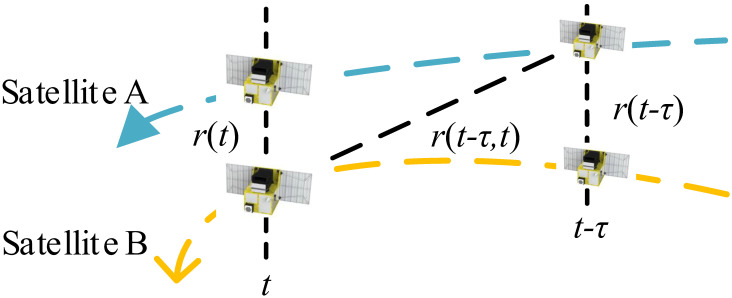
The geometric range between two satellites.

In a similar way to the analysis in [20], the following derivation will show how the satellite motion error arises when ADS-TWR is used for the time difference measurement. The relationship among the inter-satellite range, the times of flight, and the component of the satellite’s absolute speed in the direction of the line connecting satellite A to B can be deduced as follows:

(9) $$\begin{array}{l} r(t_{1}{}_{2})=r(t_{1})+v_{B12}\cdot T_{\mathrm{tof}1}\\ r(t_{34})=r(t_{3})+v_{A34}\cdot T_{\mathrm{tof}2}\\ r(t_{56})=r(t_{5})+v_{B56}\cdot T_{\mathrm{tof}3} \end{array}$$

where ${r(t}_{12})$ represents the geometric distance between the position of satellite A at moment $T_{A}{(t}_{1})$ and the position of satellite B at moment $T_{B}{(t}_{2})$, ${r(t}_{1})$ represents the distance between satellite A and B at moment $T_{A}{(t}_{1})$, and $v_{B12}$ represents the average absolute velocity in the baseline direction between satellite A and B from time $T_{A}{(t}_{1})$ to $T_{B}{(t}_{2})$, as shown in Figure 1. Other variables are named in the same way. The relationship among ${r(t}_{1})$, ${r(t}_{3})$, and ${r(t}_{5})$ is:

(10) $$\begin{array}{l} r(t_{3})=r(t_{1})+\int_{t_{1}}^{t_{3}}v_{\mathrm{AB}}(t)dt\\ r(t_{5})=r(t_{3})+\int_{t_{3}}^{t_{5}}v_{\mathrm{AB}}(t)dt \end{array}$$

where $v_{\mathrm{AB}}$ represents the instantaneous relative velocity between the two satellites. The relative motion between the satellites is visualized in Figure 3. This diagram uses r(t−τ,t) to represent ${r(t}_{12})$, ${r(t}_{34}),$ and ${r(t}_{56})$ in Equation (9), and uses r(t) to stand for ${r(t}_{1})$, ${r(t}_{3})$, and ${r(t}_{5})$.

Substituting Equations (9) and (10) into Equations (7) and (8), we obtain $M_{\Delta T}$ and $M_{\mathrm{AT}}$ as follows:

(11) $$M_{\Delta T}=\frac{1}{4}\cdot T_{\mathrm{tofAB}}\cdot \frac{v_{A34}}{c-v_{A34}}-\frac{T_{\mathrm{tofAB}}}{2K\cdot c}\cdot \left[\begin{array}{l} \left(K-N_{\mathrm{AB}}\right)\cdot \left(v_{A34}+v_{B12}\right)+\\ N_{\mathrm{AB}}\cdot \left(v_{B56}+v_{A34}\right) \end{array}\right]-\frac{v_{\mathrm{AB}35}-v_{\mathrm{AB}13}}{2\cdot c}\cdot \frac{N_{\mathrm{AB}}\cdot \left(K-N_{\mathrm{AB}}\right)\cdot T_{s}^{}}{K}$$

(12) $$M_{\mathrm{AT}}=\frac{R_{\mathrm{AB}}}{2K\cdot c}\cdot \left[\begin{array}{l} \left(K-N_{\mathrm{AB}}\right)\cdot \left(v_{A34}+v_{B12}\right)+\\ N_{\mathrm{AB}}\cdot \left(v_{B56}+v_{A34}\right) \end{array}\right]+\frac{v_{\mathrm{AB}35}-v_{\mathrm{AB}13}}{2}\cdot \frac{N_{\mathrm{AB}}\cdot \left(K-N_{\mathrm{AB}}\right)\cdot T_{s}^{}}{K}$$

where *K* is the number of time slots in one measurement period, $N_{\mathrm{AB}}$ is the number of time slots in the interval between time slot A and time slot B, $T_{s}$ is the duration of a time slot, and $v_{\mathrm{AB}13}$ and $v_{\mathrm{AB}35}$ denote the average value of $v_{\mathrm{AB}}$ in the time intervals ($t_{1}$, $t_{3}$) and ($t_{3}$, $t_{5}$), respectively (the detailed derivation is shown in Appendix B).

In fact, the direction of $v_{A34}$ is opposite to that of $v_{B12}$ and $v_{B56}$, and for a short time $v_{\mathrm{AB}35}$ and $v_{\mathrm{AB}13}$ can be considered to be equal. Therefore, the combinations of $v_{A34}{+v}_{B12}$, $v_{A34}{+v}_{B56}$ and $v_{\mathrm{AB}35}-v_{\mathrm{AB}13}$ enable ADS-TWR to reduce the motion error to a certain extent.

Equations (11) and (12) show that, compared with the ranging error, the time difference measurement error caused by satellite motion adds one error item, which is related to the transmission times and the satellites’ absolute speed.

**Figure 4:**
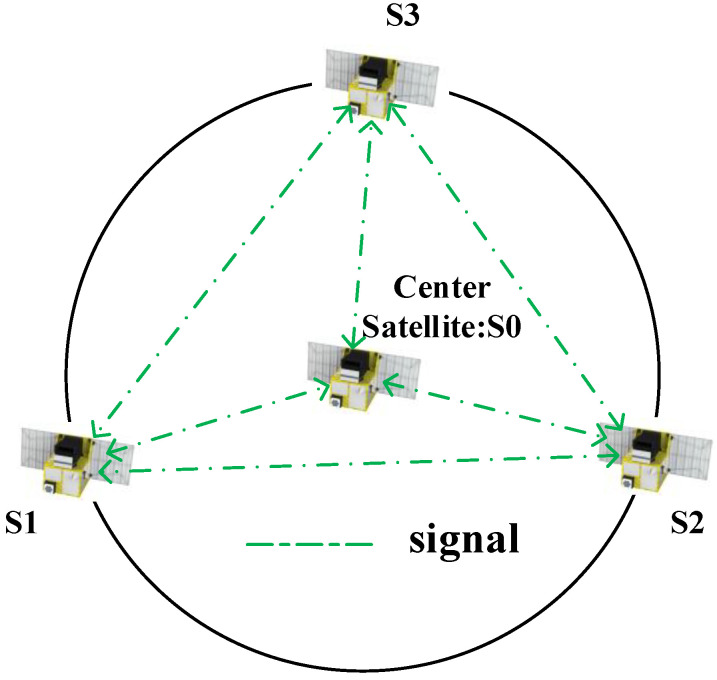
Circular formation.

The following takes a practical multi-satellite formation as an example to illustrate the influence of satellite motion. A four-satellite circular formation is simulated (S0 is a virtual node) as shown in Figure 4. The orbital elements are displayed in Table 2.

**Figure 5:**
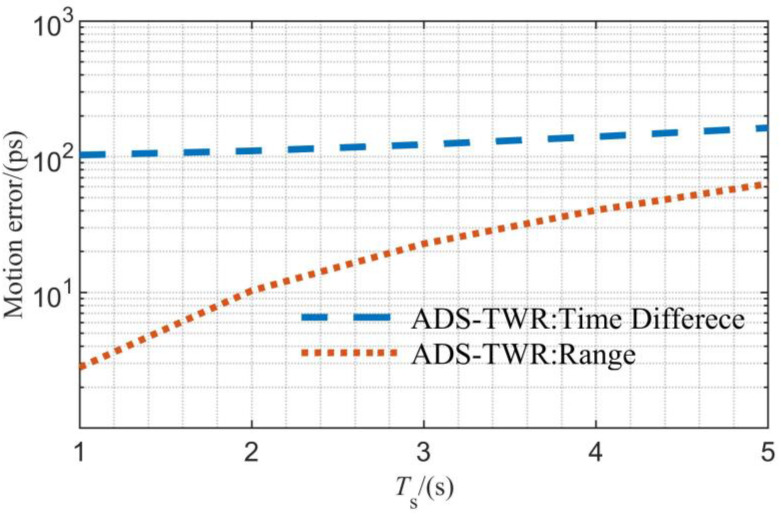
Simulation results of the motion error.

The simulation of the joint measurement error caused by satellite motion is shown in Figure 5. For the convenience of comparison, the range measurement results are normalized to time. As shown in this figure, the motion error in the time difference measurement is about 100 ps larger than that in the range measurement. However, the time difference error in the joint measurement remains at a hundred-picosecond level, which can meet the requirements of general microsatellite formations.

### 2.3. Phase Tracking Error

The phase tracking error includes the receiver thermal noise, dynamic stress, and transmitter code-phase jitter noise. For general satellite formations, such as cartwheel and circular formations, the relative dynamics among the satellites in the formation are small, and the dynamic stress error can be neglected. Therefore, we focus mainly on receiver thermal noise and transmitter code-phase jitter noise. We used a delay-locked loop (DLL) for code tracking, and the standard deviation $\sigma_{\mathrm{DLL}}$ of the receiver thermal noise error can be expressed as [20]:

(13) $$\sigma_{\mathrm{DLL}}=2\pi \sqrt{\frac{B_{L}}{2\cdot C/N_{0}}D\left(1+\frac{2}{\left(2-D\right)T_{\mathrm{coh}}C/N_{0}}\right)}\;D\geq \frac{\pi }{B_{\mathrm{fe}}T_{c}}$$

where $B_{\mathrm{fe}}$ is the double-side front-end bandwidth; $T_{c}$ is the chip period; $B_{L}$ is the single-side equivalent loop bandwidth; $T_{\mathrm{coh}}$ is the coherent integration time; *C*/*N*_0_ is the carrier–noise ratio (CNR, Carrier-to-Noise Ratio, typically in units of dBHz); and D is the early-to-late correlation spacing (in chips).

The transmitter code-phase jitter noise error $\sigma_{\theta }$ can be considered to be linear in short time slots as:

(14) $$\sigma_{\theta }=\pi \cdot f_{\mathrm{pn}}^{}\cdot T_{u}^{}\cdot \sigma_{\mathrm{Allan}}(T)$$

where $f_{\mathrm{pn}}$ is the PN code rate; $\;T_{u}$ is time slot; and $\sigma_{\mathrm{Allan}}$ is the short-term Allan deviation (ADEV) of the satellite frequency source, see [11] for a detailed analysis of the equation.

The receiver thermal noise at times *t*_1_, *t*_3_, and *t*_5_ are denoted as $\sigma_{\mathrm{DLL}1}$, $\sigma_{\mathrm{DLL}3}$, and $\sigma_{\mathrm{DLL}5}$, respectively. The transmitter code-phase jitter noise affects $T_{\mathrm{tof}2}$ with the effect denoted as $\sigma_{\theta \mathrm{tof}2}$. $n_{\Delta T}$ is the sum of the phase tracking noise errors at times $T_{A}{(t}_{4})$ and $T_{B}{(t}_{3})$, and it can be expressed as:

(15) $$n_{\Delta T}=\sqrt{\sigma_{\theta \mathrm{tof}2}^{2}+\sigma_{{}_{\mathrm{DLL}3}}^{2}}/\left(2\pi f_{\mathrm{PN}}\right)$$

**Figure 6:**
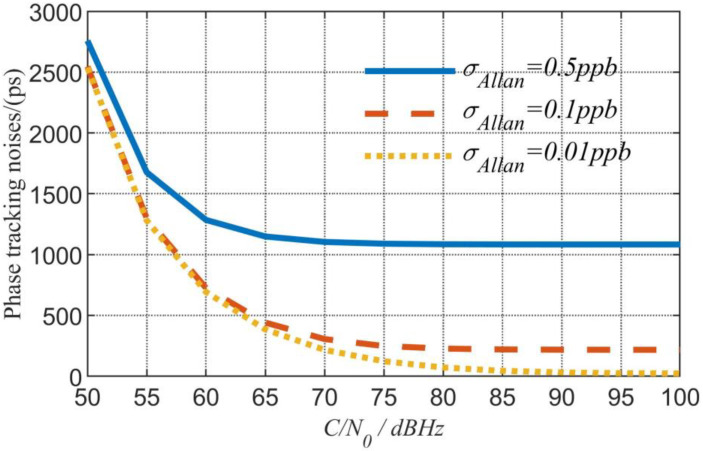
Simulation results of the phase tracking error.

The expressions of $n_{R}\;$ are available in Appendix C. The simulation result of the phase tracking error is shown in Figure 6, with the trend of the curve similar to that of the range measurement in the above reference. Under high CNR (greater than 70 dBHz), the tracking error is less than 200 ps.

### 2.4. Summary of Error Modelling

**Figure 7:**
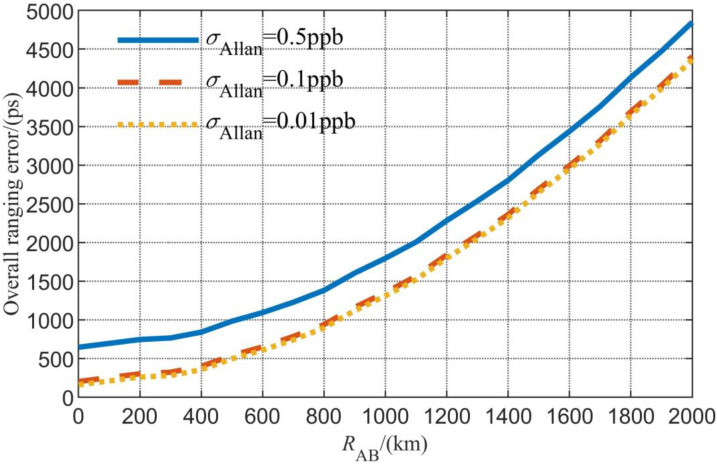
Simulation results of the overall time difference error.

The overall time difference error is shown in Figure 7. In summary, the joint measurement system has a centimeter-level accuracy for ranging and a hundred-picosecond-level accuracy for the time difference measurement.

## 3. Design of Time Difference Reference

**Figure 8:**
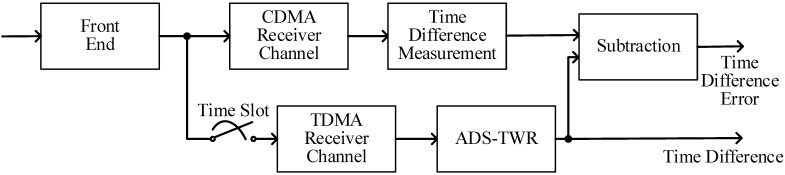
Design of the time difference reference.

This section describes the design of the time difference reference. For a two-node formation, the data from the transmitters of the two nodes modulate different PN code sequences. Each receiver of the two nodes has two independent receiving channels which receive the TDMA signal and CDMA signal, respectively, as shown in Figure 8. The CDMA channel keeps tracking the received signal and calculates the time difference between the two nodes using the time difference measurement method proposed in [11]. The TDMA channel works only in the time slot of the node and measures the range and time difference using ADS-TWR.

Different from TDMA, the CDMA-based time difference measurement is temporally continuous. Thus, the frequency deviation caused error and motion caused error are small and the total measurement error of time difference measurement using CDMA is much less than that using TDMA. In a ground static environment, the motion error is zero for both CDMA and TDMA; however, the phase tracking noise is related to the loop bandwidth of the delay-locked loop (DLL) [20], and the total measurement error of CDMA can be controlled much less than that of TDMA by decreasing the loop bandwidth of CDMA. Therefore, the reference designed as such can be used for the experimental verification of the time difference measurement performance of the proposed joint measurement method. Because the CDMA system is realized on the same hardware as the TDMA joint measurement system, the overall system had the prominent advantages of simplicity and miniaturization.

## 4. Clock Synchronization Scheme

### 4.1. Multi-Satellite Clock Synchronization

**Figure 9:**
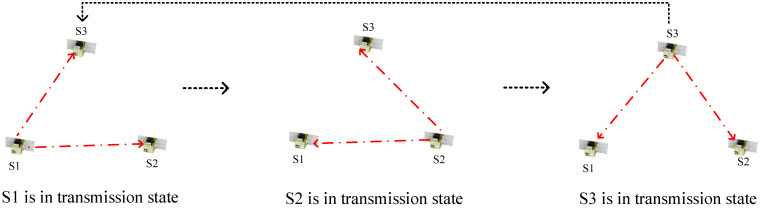
Clock synchronization scheme.

Considering the advantages and disadvantages of the NTP and the consensus clock synchronization schemes, as well as the distributed multi-satellite measurement scheme in [10], we propose an innovative clock synchronization scheme which is shown in Figure 9. The synchronization process can be described as follows: each satellite only transmits a signal in its own time slot and receives other satellites’ signals in the other time slots. Take three satellite (numbered S1, S2, S3) formations as an example. When S1 is in the transmission state, the remaining two satellites in the formation measure the time difference between themselves and S1 based on the joint measurement method, thus completing clock synchronization with S1. When the time slot of S1 ends, satellite S2 switches into the transmission state, so S2 becomes the new root node, and the remaining two satellites synchronize with S2. In the end, clock synchronization is established and maintained among all satellites in the formation.

**Figure 10:**
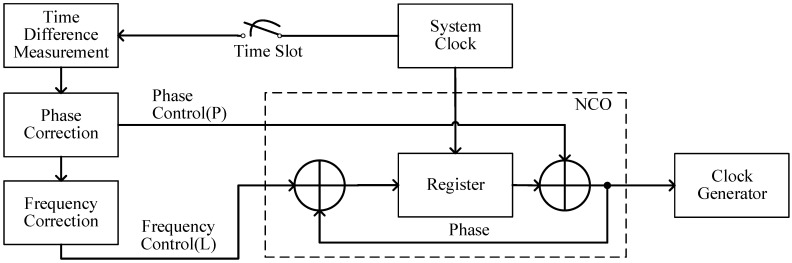
Clock synchronization process.

Similar to the consensus clock synchronization scheme, the new clock synchronization is divided into two steps as shown in Figure 10. First, each satellite measures the time difference between itself and the satellite in the transmission state and corrects the clock phase by modifying the NCO (numerically controlled oscillator) phase register. Second, the frequency difference between the two satellites is obtained by calculating the difference between the current and last time difference measurements and dividing it by the measurement cycle time, which can be compensated by modifying the frequency control word of the NCO.

### 4.2. Clock Synchronization Performance Analysis

The phase correction error depends on the time difference measurement error. Since a hundred-picosecond-level accuracy of time difference measurement can be achieved, the same level of accuracy can also be expected for phase correction.

We start with the case that the clock frequency is not corrected. The maximum synchronization error can be expressed as:

(16) $$T_{\mathrm{syn}\_\mathrm{error}}=T_{\mathrm{update}}\cdot K_{A}-T_{\mathrm{update}}\cdot K_{B}$$

where $T_{\mathrm{update}}$ is the synchronization cycle equal to 5 s and the frequency accuracy is 0.01 ppm. According to Equation (16), when satellite A is in the transmission state, the maximum synchronization error $T_{\mathrm{syn}\_\mathrm{error}}$ of satellite B will reach hundreds of nanoseconds. Thus, frequency correction is necessary in order to achieve high-precision inter-satellite clock synchronization.

Frequency correction is performed by calculating the ratio of the frequencies of the two satellites and feeding the result to the frequency control word of the NCO. The modified parameters are calculated as in Equation (17).

(17) $$\Delta f(i)=\frac{\Delta T(i)-\Delta T(i-1)}{T_{\mathrm{update}}}\cdot f_{\mathrm{local}}+\Delta f_{\mathrm{NCO}}$$

where Δf(i) is the frequency difference at time i, ΔT(i) is the time difference measurement at time i, $f_{\mathrm{local}}$ is the local clock frequency of the satellite in the receiving state, and $\Delta f_{\mathrm{NCO}}$ is the NCO frequency control word error. Combining Equations (16) and (17), we can obtain the expression of the synchronization error after frequency correction as shown in Equation (18).

(18) $$T_{\mathrm{syn}\_\mathrm{error}}=T_{\mathrm{update}}\cdot K_{A}-T_{\mathrm{update}}\cdot (K_{B}+\frac{\Delta f(i)}{f_{0}})$$

where $f_{0}$ is the nominal frequency equal to 40 MHz. The frequency correction error calculated in Equation (18) is affected by three factors. The first one is the frequency source stability, and its effect is only tens of picoseconds when the frequency stability is 0.01 ppb, according to Equation (16). The second one is the time difference measurement error of the joint measurement. When the parameters are used as in Section 2, the difference of the time difference measurement error at time i and i−1 is about 317 ps, which will bring a frequency correction error of 2.5 mHz according to Equation (17). Substituting Δf(i) in Equation (18) with 2.5 mHz will result in a synchronization error of 317 ps as well, since $f_{\mathrm{local}}$ and $f_{0}$ are almost the same. The third one is the NCO frequency word error. The worst error of the frequency control word in this paper is 9.313 MHz. By substituting it into Equation (18), we find that the synchronization error incurred by the NCO frequency word error is 1.1641 ns. In summary, the worst synchronization error can reach about 2 ns after frequency correction.

### 4.3. Simulation of Multi-Satellite Clock Synchronization Scheme

The simulation parameters are displayed in Table 3, where $K_{i}$ represents the frequency accuracy of satellite i; ${\mathrm{eNCO}}_{i}$ represents the NCO frequency word error of satellite i; and ${\mathrm{eTD}}_{\mathrm{ij}}$ represents the standard deviation of the time difference measurement error between satellite i and j. Take the clock synchronization between satellites 1 and 2 as an example, $K_{1}$ and $K_{2}$ in Table 3 represent $K_{A}$ and $K_{B}$ in Equation (18), respectively. Additionally, ${(\mathrm{eNCO}}_{1}-{\mathrm{eNCO}}_{2})$ is equivalent to $\Delta f_{\mathrm{NCO}}$ in Equation (17), and ${\mathrm{eTD}}_{12}$ corresponds to the standard deviation of the errors of ΔT(i) and ΔT(i−1).

**Figure 11:**
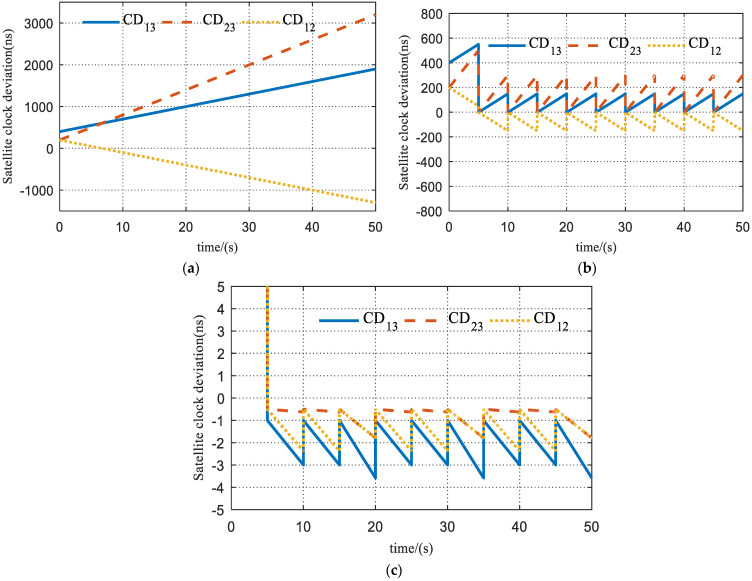
Simulation results of inter-satellite clock synchronization: (**a**) satellite clock deviation without synchronization; (**b**) satellite clock deviation with only phase correction; and (**c**) satellite clock deviation with both phase and frequency correction.

The simulation results of the performance of the clock synchronization scheme are shown in Figure 11. During the first time slot (0~5 s), clock synchronization has not been established, so the clock deviation is more obvious. The initial clock time of satellite 1, satellite 2, and satellite 3 are $5.12+2\times 10^{-7}$ s, 5.12 s, and $5.12-2\times 10^{-7}$ s, respectively.

Figure 11a shows the clock deviation between Satellites 1 and 3 (noted as CD_13_), Satellites 2 and 3 (noted as CD_23_), and Satellites 1 and 2 (noted as CD_12_) without phase correction and frequency correction for each satellite. Figure 11b shows the clock deviation with phase correction only. It can be seen that there is still a hundred-ns level gap among the satellite clocks due to the absence of frequency error correction. Figure 11c shows the clock deviation with both phase correction and frequency correction, which remains at only an ns level, demonstrating the best synchronization performance.

## 5. Experimental Verification

### 5.1. Experimental Platform

**Figure 12:**
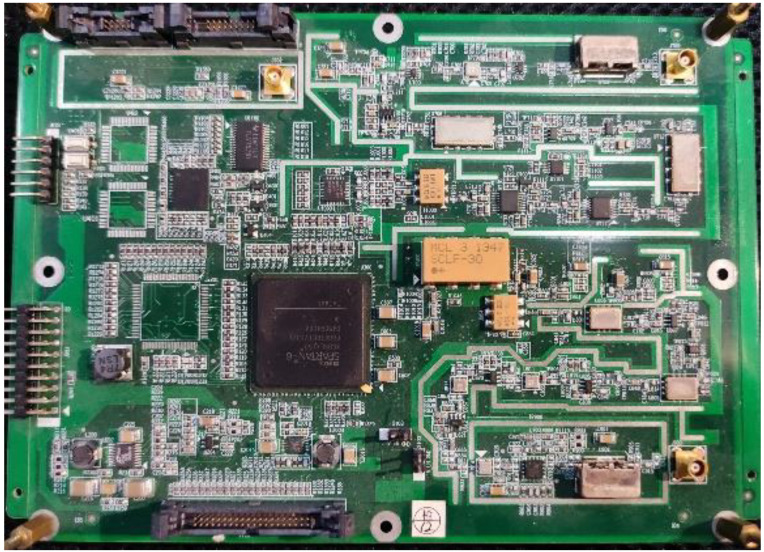
Prototype of the RF measurement transceiver.

An inter-satellite RF measurement transceiver was designed and implemented, based on which the measurement system was constructed. The S-band RF measurement transceiver consisted of a digital signal processing module and two RF front-ends for reception and transmission, respectively. Since the system is based on the TDMA mechanism, the receiver and transmitter operate on the same carrier frequency and can be freely switched on and off. Figure 12 shows a prototype of the RF measurement transceiver.

**Figure 13:**
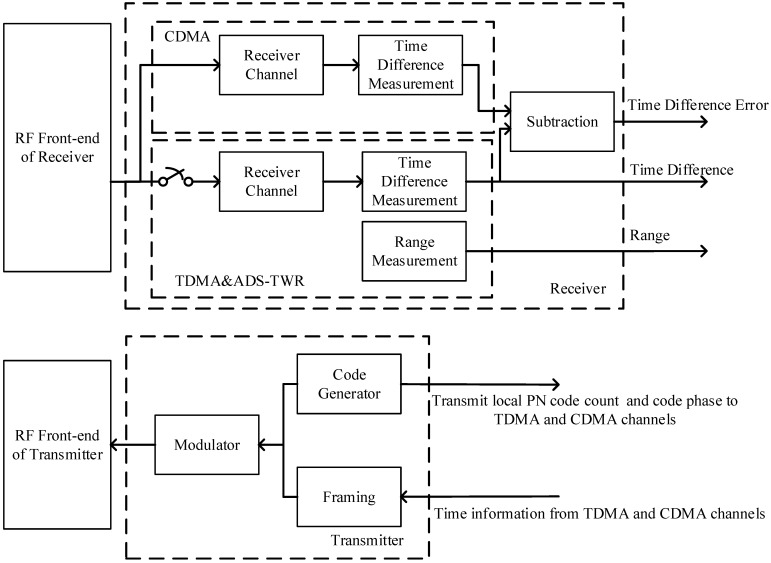
Joint measurement module.

The measurement processing module of the transceiver was developed based on an FPGA (field programmable gate array). The structure of the processing module is displayed in Figure 13 and its main specifications are listed in Table 4. The local PN code count and phase can be extracted from the local PN code generator in the transmitter to form the signal reception time. The TDMA receiving channel obtains the received PN code chip count and phase through the tracking loop to form the signal transmission time and then obtains the range and time difference measurements by using the ADS-TWR method.

### 5.2. Two-Node Experiment

**Figure 14:**
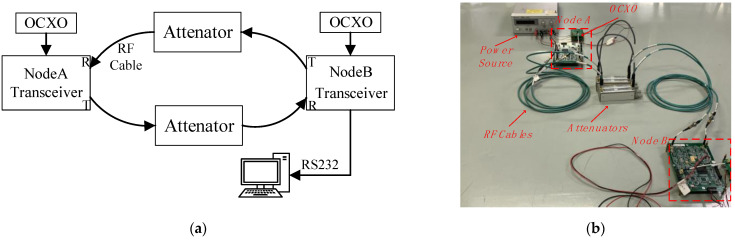
Test platform: (**a**) test platform concept; (**b**) test platform photo.

The test platform consists of two RF transceivers and other auxiliary devices including miniaturized oven-controlled crystal oscillators (OCXOs), RF cables, attenuators, an RS232 cable, and a computer. As shown in Figure 14, the attenuator was connected to both transceivers via RF cables to adjust the signal strength and protect the transceivers. The results of the joint measurement and clock synchronization were sent to the computer via the RS232. The transmission speed in the RF cable needs to be calibrated before performance evaluation, and the calibration result was 20,660,000 m/s. At the same time, the hardware delay could be calculated as 3.294 μs.

#### 5.2.1. Joint Measurement Experimental Verification

**Figure 15:**
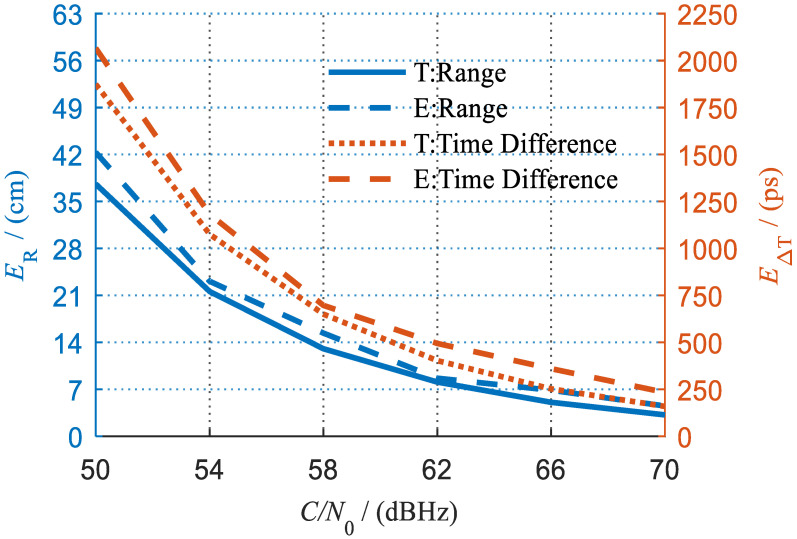
Experimental results (T means theory, E means experiment).

After compensating for the hardware delays, we obtained the overall measurement errors compared with the theoretical values as shown in Figure 15. It can be seen that the experimental accuracy is well consistent with the theoretical analysis. At a high carrier-to-noise ratio of 70 dBHz, an overall ranging error of 4.52 cm and a time difference measurement error of 233 ps can be achieved.

It is noteworthy that, as revealed by the preceding analysis, the error of time difference measurement should be equal to that of ranging in a ground static environment. However, the former seems larger than the latter, as shown in Figure 15. For example, the time difference measurement error and normalized ranging error are 233 ps and 151 ps, respectively, at a carrier-to-noise ratio of 70 dBHz. This is because the measurement signals were transmitted in RF cables rather than in a vacuum. They are actually equivalent when the calculation is performed with the transmission speed in RF cables.

#### 5.2.2. Clock Synchronization Experimental Verification

**Figure 16:**
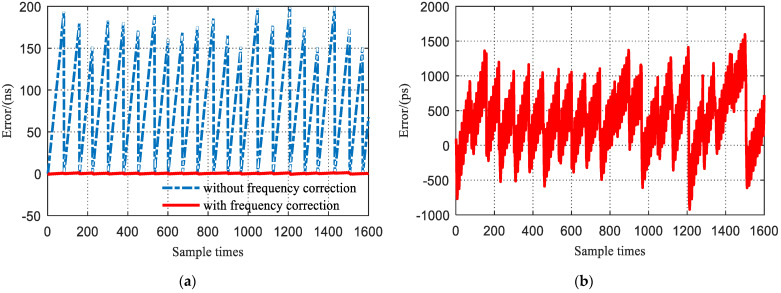
Experimental clock synchronization results: (**a**) synchronization error with frequency correction vs. without; (**b**) detailed view of the synchronization error with frequency correction (standard deviation = 431.60 ps).

Figure 16a compares the clock synchronization error with and without frequency correction. Apparently, the synchronization error between two phase corrections without frequency correction can reach hundreds of nanoseconds, which is much larger than that with frequency correction. Figure 16b is a magnified display of the clock synchronization results with frequency correction. It can be seen that the maximum error between two phase corrections is only about 1 ns, which is in accordance with the theoretical analysis.

### 5.3. Multi-Node Experiment

**Figure 17:**
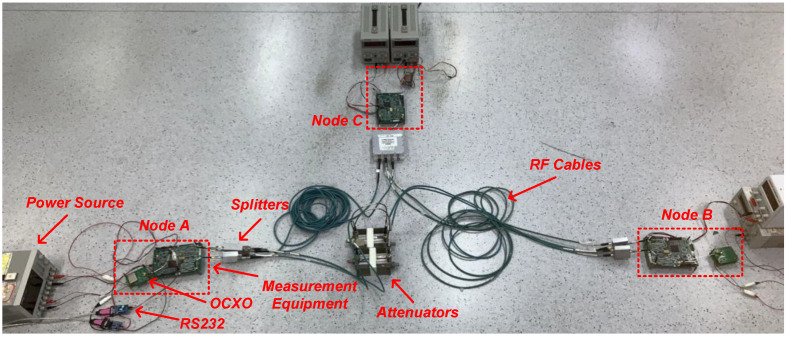
Experimental platform.

In [10] It was demonstrated that the measurement accuracy hardly deteriorated as the number of nodes increased, even when the number of nodes reached tens. A three-node experimental platform was established, as shown in Figure 17, to verify the performance and scalability of the proposed joint RF measurement method and clock synchronization scheme for multi-microsatellite formations.

The experimental platform was constructed as follows: the three nodes were connected with fixed attenuators and splitters. Node A and node B were connected with 3-m-long RF cables, node A and node C were connected with 1-m-long RF cables, and node B and node C were connected with 5-m-long RF cables.

#### 5.3.1. Joint Measurement Experimental Verification

**Figure 18:**
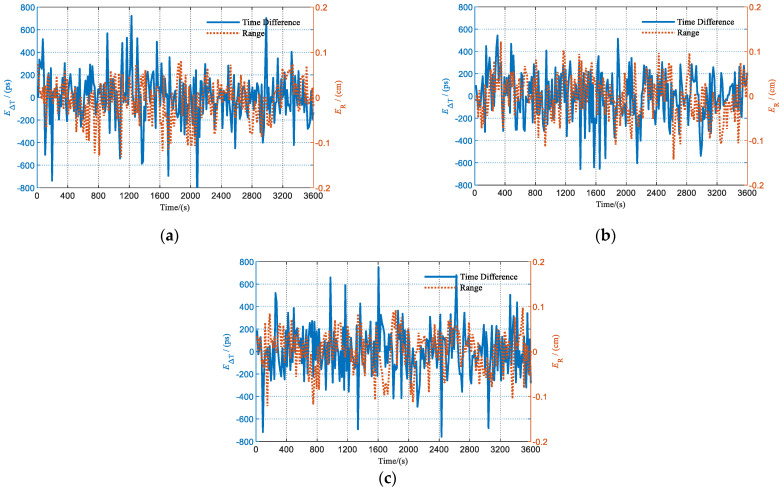
Multi-node joint measurement experimental results: (**a**) measurements between node A and B; (**b**) measurements between node A and C; and (**c**) measurements between node B and C.

The test results of the joint measurement experiment are shown in Figure 18. After the circuit delay was compensated, the standard deviations of the errors of the range and time difference measurements between node A and node B were 4.49 cm and 224.29 ps, respectively. The standard deviations of the errors of the range and time difference measurements between node A and node C were 4.64 cm and 217.03 ps, respectively. The standard deviations of the errors of the range and time difference measurements between node B and node C were 4.49 cm and 226.59 ps, respectively. The above results show that with the increase in the number of nodes, there is no significant degradation of the measurement performance, which accords with the theoretical analysis and validates the scalability of the proposed joint measurement method.

#### 5.3.2. Clock Synchronization Experimental Verification

**Figure 19:**
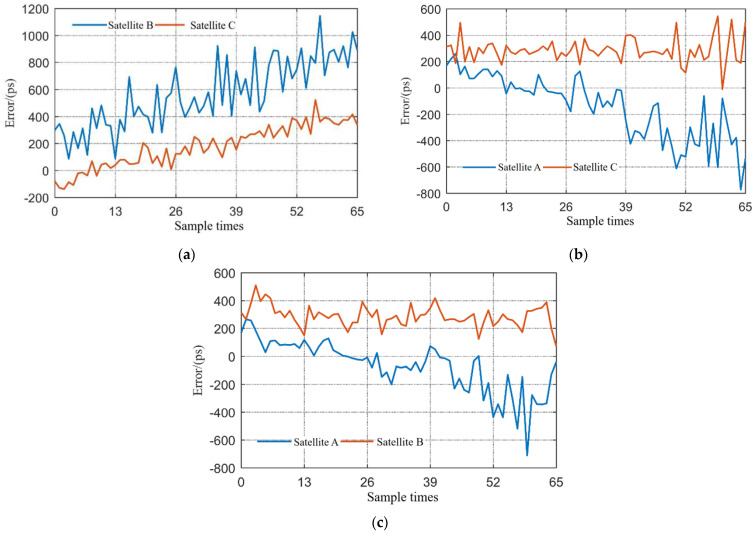
Multi-node clock synchronization experimental results: (**a**) results of node B and C when node A is the master node; (**b**) results of node A and C when node B is the master node; and (**c**) results of node A and B when node C is the master node.

Since each satellite performs clock synchronization independently, the results of multi-node clock synchronization are similar to that of two-node clock synchronization. Figure 19 shows the clock synchronization errors when different satellite nodes acted as the master nodes, i.e., when different satellites were in the transmission state. The sampling period was 10 s. It can be seen that in the multi-node case, the system can still work well and maintain synchronization errors of less than 1 nanosecond.

## 6. Conclusions

In this study, a method was proposed for the joint RF measurement of inter-satellite range and time difference for multi-satellite formations. Based on this method, a new clock synchronization scheme was proposed. The experimental results showed that the joint measurement system had a centimeter-level accuracy for ranging and a hundred-picosecond-level accuracy for time difference measurement, thus verifying the correctness of the theoretical measurement model. The experimental results also demonstrated that the maximum clock synchronization error was only about 1 ns. This research effectively solves the existing problem in the literature that a highly accurate inter-satellite range and time difference measurement cannot be obtained simultaneously using only one piece of equipment and provides a foundation for the miniaturized and high-precision RF navigation of multi-satellite formations.

## Figures and Tables

**Table 1:** Parameter setup.

Parameters	Value
Double-side front-end bandwidth/MHz	20
Single-side equivalent bandwidth/Hz	35
Carrier-to-noise ratio/dBHz	70
Loop order	2
PN code rate/MHz	5.115
Early-to-late correlation spacing/chip	1
Coherent integration time/μs	50
Time slot/s	5

**Table 2:** Orbital elements.

Orbital Elements	S0	S1	S2	S3
a/km	6878.14	6878.14	6878.14	6878.14
*e*	0	0.0087	0.0087	0.0087
i/°	97	96.9992	97.7495	96.2501
Ω/°	0	0.8722	359.5632	359.5646
w/°	0	180.1063	59.9439	299.9498
$M_{0}$/°	0	0.0087	0.0087	0.0087

a, *e*, i, Ω, w, and $M_{0}$ represent the semi-major axis, eccentricity, inclination, longitude of ascending node, argument of periapsis, and mean anomaly, respectively.

**Table 3:** Simulation parameters.

Simulation Parameters	Value	Simulation Parameters	Value
$K_{1}$	$1+2\times 10^{-8}$	${\mathrm{eTD}}_{12}/s$	$5\times 10^{-10}$
$K_{2}$	$1+5\times 10^{-8}$	${\mathrm{eTD}}_{13}/s$	$1\times 10^{-9}$
$K_{3}$	$1-1\times 10^{-8}$	${\mathrm{eTD}}_{21}/s$	$-5\times 10^{-10}$
${\mathrm{eNCO}}_{1}/\mathrm{Hz}$	$4.65\times 10^{-3}$	${\mathrm{eTD}}_{23}/s$	$5\times 10^{-10}$
${\mathrm{eNCO}}_{2}/\mathrm{Hz}$	$-4.65\times 10^{-3}$	${\mathrm{eTD}}_{31}/s$	$-1\times 10^{-9}$
${\mathrm{eNCO}}_{3}/\mathrm{Hz}$	0	${\mathrm{eTD}}_{32}/s$	$-5\times 10^{-10}$

**Table 4:** Main specifications of the RF measurement transceiver.

Parameters	Value
Nominal frequency of the frequency source/MHz	40
Stability of the frequency source	1 × 10^−11^
Accuracy of the frequency source	1 × 10^−8^
RF frequency/MHz	2352
Transmit power/mW	400
Receiver acquisition sensitivity/dBm	−112
Receiver dynamic range/dB	≥60

## Data Availability

The data presented in this study are available on request from the corresponding author.

## Appendix A

The measurement delays include the ionosphere delay and hardware delay. The descriptions in Appendix A can all be found in [20] and are listed here for the convenience of the reader. Considering the above factors, $T_{\mathrm{rdA}}$, $T_{\mathrm{rdB}}$, $T_{\mathrm{reA}}$, and $T_{\mathrm{reB}}$ can be replaced by ${\hat{T}}_{\mathrm{rdA}}$, ${\hat{T}}_{\mathrm{rdB}}$, ${\hat{T}}_{\mathrm{reA}}$, and ${\hat{T}}_{\mathrm{reB}}$, respectively, which can be expressed as [20]:

(A1) $$\begin{array}{l} {\hat{T}}_{\mathrm{rdA}}=T_{\mathrm{rdA}}\cdot K_{A}+n_{\mathrm{rdA}}\\ =[\left(T_{\mathrm{tof}1}+\tau_{\mathrm{io}1}+\tau_{\mathrm{dtA}}+\tau_{\mathrm{drB}}\right)+T_{\mathrm{reB}}\left.+\left(T_{\mathrm{tof}2}+\tau_{\mathrm{io}2}+\tau_{\mathrm{dtB}}+\tau_{\mathrm{drA}}\right)\right]\cdot K_{A}+n_{\mathrm{rdA}}\\ {\hat{T}}_{\mathrm{reA}}=T_{\mathrm{reA}}\cdot K_{A}+n_{\mathrm{reA}}\\ {\hat{T}}_{\mathrm{rdB}}=T_{\mathrm{rdB}}\cdot K_{B}+n_{\mathrm{rdB}}\\ =\left[\left(T_{\mathrm{tof}2}+\tau_{\mathrm{io}2}+\tau_{\mathrm{dtB}}+\tau_{\mathrm{drA}}\right)+T_{\mathrm{reA}}\right.\left.+\left(T_{\mathrm{tof}3}+\tau_{\mathrm{io}3}+\tau_{\mathrm{dtA}}+\tau_{\mathrm{drB}}\right)\right]\cdot K_{B}+n_{\mathrm{rdB}}\\ {\hat{T}}_{\mathrm{reB}}=T_{\mathrm{reB}}\cdot K_{B}+n_{\mathrm{reB}} \end{array}$$

where $K_{A}$ and $K_{B}$ represent the ratios of the onboard clock frequency to the nominal frequency for satellites A and B, respectively. $n_{\mathrm{rdA}}$, $n_{\mathrm{rdB}}$, $n_{\mathrm{reA}}$, and $n_{\mathrm{reB}}$ are the phase tracking errors of ${\hat{T}}_{\mathrm{rdA}}$, ${\hat{T}}_{\mathrm{rdB}}$, ${\hat{T}}_{\mathrm{reA}}$, and ${\hat{T}}_{\mathrm{reB}}$, respectively, and $\tau_{\mathrm{io}1}$, $\tau_{\mathrm{io}2}$, and $\tau_{\mathrm{io}3}$ represent the ionospheric delays. $\tau_{\mathrm{dtA}}$ and $\tau_{\mathrm{dtB}}$ are the hardware delays of the transmitter circuit; $\tau_{\mathrm{drA}}$ and $\tau_{\mathrm{drB}}$ are the hardware delays of the receiver circuit.

Before computing an ADS-TWR measurement result, ${\hat{T}}_{\mathrm{rdA}}$ and ${\hat{T}}_{\mathrm{rdB}}\;$ need to be preprocessed to eliminate the impact of the ionospheric delay and hardware delay. After compensation, ${\hat{T}}_{\mathrm{rdA}}$ and ${\hat{T}}_{\mathrm{rdB}}$ are obtained as follows [20]:

(A2) $$\begin{array}{l} {\hat{T}}_{\mathrm{rdA}}=\left(T_{\mathrm{tof}1}+T_{\mathrm{tof}2}+T_{\mathrm{reB}}\right)\cdot K_{A}+\varepsilon_{\mathrm{rdA}}+n_{\mathrm{rdA}}\\ {\hat{T}}_{\mathrm{rdB}}=\left(T_{\mathrm{tof}2}+T_{\mathrm{tof}3}+T_{\mathrm{reA}}\right)\cdot K_{B}+\varepsilon_{\mathrm{rdB}}+n_{\mathrm{rdB}} \end{array}$$

where $\varepsilon_{\mathrm{rdA}}$ and $\varepsilon_{\mathrm{rdB}}$ are the residual errors of the hardware delay and ionospheric delay, respectively, which are very small and can be neglected.

The ionospheric delay $\tau_{\mathrm{io}}$ can be expressed as [20]:

(A3) $$\tau_{io}=40.28N_{e}/(c\cdot f^{2})$$

where $N_{e}$ represents the electron density and f is the frequency of the signal.

Dual-frequency ionosphere correction is an effective technique for the elimination of ionospheric delay. The approach can be briefly explained as follows. As the ionospheric delay is related to the frequency, satellites can transmit measurement signals on two different frequencies and obtain two sets of ranging results, which are linearly combined to eliminate the first-order error term of the ionospheric delay. The remaining effect due to higher-order terms is very small and generally ignored.

Hardware delay is a system deviation that can be compensated through calibration. The calibration process is described as follows. The range is measured a certain number of times under a stationary condition. Then, the average of these ranging values ${\bar{R}}_{\mathrm{static}}$ can be obtained as [20]:

(A4) $${\bar{R}}_{\mathrm{static}\;}=\sum_{i=1}^{N}{\left(R_{\mathrm{static}\;}\right)}_{i}/N_{\mathrm{static}\;}=T_{\mathrm{tof}\;}\cdot \frac{K_{A}+K_{B}}{2}+\tau_{d}\cdot \frac{K_{A}+K_{B}}{4}$$

where $R_{\mathrm{static}}$ represents a single measurement result, $N_{\mathrm{static}}$ represents the number of measurements, and $\tau_{d}$ is the hardware delay. Combined with the time of flight, the hardware delay parameter $R_{d}$ can be computed as [20]:

(A5) $$R_{d}=\left({\bar{R}}_{\mathrm{static}\;}-T_{\mathrm{tof}\;}\right)\times 2=\tau_{d}\cdot \left(K_{A}+K_{B}\right)/2+T_{\mathrm{tof}\;}\cdot \left(K_{A}+K_{B}-2\right)$$

As the hardware delay is a system deviation, the hardware delay parameter can also be applied in dynamic scenes.

## Appendix B

Equation (9) can be rewritten as follows:

(A6) $$\begin{array}{l} r(t_{1}{}_{2})=c*T_{\mathrm{tof}1}=r(t_{1})+v_{B12}\cdot T_{\mathrm{tof}1}\\ r(t_{34})=c*T_{\mathrm{tof}2}=r(t_{3})+v_{A34}\cdot T_{\mathrm{tof}2}\\ r(t_{56})=c*T_{\mathrm{tof}3}=r(t_{5})+v_{B56}\cdot T_{\mathrm{tof}3} \end{array}$$

Equation (10) can be rewritten as follows:

(A7) $$\begin{array}{l} r(t_{1})=r(t_{3})-\int_{t_{1}}^{t_{3}}v_{\mathrm{AB}}(t)dt=c*T_{\mathrm{tofAB}}-\int_{t_{1}}^{t_{3}}v_{\mathrm{AB}}(t)dt\\ r(t_{5})=r(t_{3})+\int_{t_{3}}^{t_{5}}v_{\mathrm{AB}}(t)dt=c*T_{\mathrm{tofAB}}+\int_{t_{3}}^{t_{5}}v_{\mathrm{AB}}(t)dt \end{array}$$

Thus, $T_{\mathrm{tof}1}$, $T_{\mathrm{tof}2}$ and $T_{\mathrm{tof}3}\;$ can be deduced as follows:

(A8) $$\begin{array}{l} T_{\mathrm{tof}1}=\frac{r(t_{1})}{c-v_{B12}}=\frac{c*T_{\mathrm{tofAB}}-\int_{t_{1}}^{t_{3}}v_{\mathrm{AB}}(t)dt}{c-v_{B12}}\\ T_{\mathrm{tof}3}=\frac{r(t_{5})}{c-v_{B56}}=\frac{c*T_{\mathrm{tofAB}}+\int_{t_{3}}^{t_{5}}v_{\mathrm{AB}}(t)dt}{c-v_{B56}}\\ T_{\mathrm{tof}2}=\frac{c*T_{\mathrm{tofAB}}}{c-v_{A34}} \end{array}$$

Substituting $T_{\mathrm{tof}1}$, $T_{\mathrm{tof}2}$, and $T_{\mathrm{tof}3}$ into Equation (7), the ADS-TWR motion error for ranging can be expressed as:

(A9) $$\begin{array}{l} M_{\mathrm{AT}}=\frac{c}{4}\cdot \left(T_{\mathrm{tof}1}+T_{\mathrm{tof}2}-2T_{\mathrm{tofAB}}\right)\cdot K_{A}+\frac{c}{4}\cdot \left(T_{\mathrm{tof}2}+T_{\mathrm{tof}3}-2T_{\mathrm{tofAB}}\right)\cdot K_{B}+\frac{c}{4}\cdot \left(T_{\mathrm{tof}3}-T_{\mathrm{tof}1}\right)\cdot \left(\frac{T_{\mathrm{reB}}\cdot K_{A}}{T_{\mathrm{tof}2}+T_{\mathrm{tof}3}+T_{\mathrm{reA}}+T_{\mathrm{reB}}}-\frac{T_{\mathrm{reA}}\cdot K_{B}}{T_{\mathrm{tof}1}+T_{\mathrm{tof}2}+T_{\mathrm{reB}}+T_{\mathrm{reA}}}\right)\\ =\frac{c}{4}\cdot \left(T_{\mathrm{tof}1}+T_{\mathrm{tof}2}-2T_{\mathrm{tofAB}}\right)\cdot K_{A}+\frac{c}{4}\cdot \left(T_{\mathrm{tof}2}+T_{\mathrm{tof}3}-2T_{\mathrm{tofAB}}\right)\cdot K_{B}+\frac{c}{4}\cdot \left(T_{\mathrm{tof}3}-T_{\mathrm{tof}1}\right)\cdot \gamma \\ =\frac{c}{4}K_{A}(\frac{c*T_{\mathrm{tofAB}}-\int_{t_{1}}^{t_{3}}v_{\mathrm{AB}}(t)dt}{c-v_{B12}}+\frac{c*T_{\mathrm{tofAB}}}{c-v_{A34}}-2T_{\mathrm{tofAB}})+\frac{c}{4}K_{B}\cdot \left(\frac{c*T_{\mathrm{tofAB}}}{c-v_{A34}}+\frac{c*T_{\mathrm{tofAB}}+\int_{t_{3}}^{t_{5}}v_{\mathrm{AB}}(t)dt}{c-v_{B56}}-2T_{\mathrm{tofAB}}\right)\\ +\frac{c}{4}\gamma (\frac{c*T_{\mathrm{tofAB}}+\int_{t_{3}}^{t_{5}}v_{\mathrm{AB}}(t)dt}{c-v_{B56}}-\frac{c*T_{\mathrm{tofAB}}-\int_{t_{1}}^{t_{3}}v_{\mathrm{AB}}(t)dt}{c-v_{B12}})\\ =\frac{c}{4}T_{\mathrm{tofAB}}K_{A}(\frac{c-\frac{\int_{t_{1}}^{t_{3}}v_{\mathrm{AB}}(t)dt}{T_{\mathrm{tofAB}}}}{c-v_{B12}}+\frac{c}{c-v_{A34}}-2)+\frac{c}{4}T_{\mathrm{tofAB}}K_{B}\cdot \left(\frac{c}{c-v_{A34}}+\frac{c+\frac{\int_{t_{3}}^{t_{5}}v_{\mathrm{AB}}(t)dt}{T_{\mathrm{tofAB}}}}{c-v_{B56}}-2\right)+\frac{c}{4}T_{\mathrm{tofAB}}\gamma (\frac{c+\frac{\int_{t_{3}}^{t_{5}}v_{\mathrm{AB}}(t)dt}{T_{\mathrm{tofAB}}}}{c-v_{B56}}-\frac{c-\frac{\int_{t_{1}}^{t_{3}}v_{\mathrm{AB}}(t)dt}{T_{\mathrm{tofAB}}}}{c-v_{B12}})\\ =\frac{c}{4}T_{\mathrm{tofAB}}K_{A}(\frac{c}{c-v_{B12}}+\frac{c}{c-v_{A34}}-2)+\frac{c}{4}T_{\mathrm{tofAB}}K_{B}\cdot \left(\frac{c}{c-v_{A34}}+\frac{c}{c-v_{B56}}-2\right)+\frac{c}{4}T_{\mathrm{tofAB}}\gamma (\frac{c}{c-v_{B56}}-\frac{c}{c-v_{B12}})\\ \begin{array}{l} -\frac{c}{4}K_{A}\frac{\int_{t_{1}}^{t_{3}}v_{\mathrm{AB}}(t)dt}{c-v_{B12}}+\frac{c}{4}K_{B}\frac{\int_{t_{3}}^{t_{5}}v_{\mathrm{AB}}(t)dt}{c-v_{B56}}+\frac{c}{4}\gamma (\frac{\int_{t_{3}}^{t_{5}}v_{\mathrm{AB}}(t)dt}{c-v_{B56}}+\frac{\int_{t_{1}}^{t_{3}}v_{\mathrm{AB}}(t)dt}{c-v_{B12}})\\ =\frac{c}{4}T_{\mathrm{tofAB}}K_{A}(\frac{c}{c-v_{B12}}+\frac{c}{c-v_{A34}}-2)+\frac{c}{4}T_{\mathrm{tofAB}}K_{B}\cdot \left(\frac{c}{c-v_{A34}}+\frac{c}{c-v_{B56}}-2\right)+\frac{c}{4}T_{\mathrm{tofAB}}\gamma (\frac{c}{c-v_{B56}}-\frac{c}{c-v_{B12}}) \end{array}\\ -(\frac{c}{4}K_{A}-\frac{c}{4}\gamma )\frac{\int_{t_{1}}^{t_{3}}v_{\mathrm{AB}}(t)dt}{c-v_{B12}}+(\frac{c}{4}K_{B}+\frac{c}{4}\gamma )\frac{\int_{t_{3}}^{t_{5}}v_{\mathrm{AB}}(t)dt}{c-v_{B56}} \end{array}$$

where *γ* is defined as:

(A10) $$\begin{array}{l} \gamma =\frac{T_{\mathrm{reB}}\cdot K_{A}}{T_{\mathrm{tof}2}+T_{\mathrm{tof}3}+T_{\mathrm{reA}}+T_{\mathrm{reB}}}-\frac{T_{\mathrm{reA}}\cdot K_{B}}{T_{\mathrm{tof}1}+T_{\mathrm{tof}2}+T_{\mathrm{reB}}+T_{\mathrm{reA}}}\\ =\frac{T_{\mathrm{reB}}\cdot K_{A}}{\left[T_{\mathrm{tofAB}}\cdot c/\left(c-v_{A34}\right)+T_{\mathrm{tofAB}}\cdot c/\left(c-v_{B56}\right)+\int_{t_{3}}^{t_{5}}v_{\mathrm{AB}}\left(t\right)dt/\left(c-v_{B56}\right)+T_{\mathrm{reA}}+T_{\mathrm{reB}}\right]}\\ \;-\frac{T_{\mathrm{reA}}\cdot K_{B}}{\left[T_{\mathrm{tofAB}}\cdot c/\left(c-v_{B12}\right)-\int_{t_{1}}^{t_{3}}v_{\mathrm{AB}}\left(t\right)dt/\left(c-v_{B12}\right)+T_{\mathrm{tofAB}}\cdot c/\left(c-v_{A34}\right)+T_{\mathrm{reB}}+T_{\mathrm{reA}}\right]} \end{array}$$

The simplification process of Equation (A9) is shown in Equation (A11).

(A11) $$\begin{array}{l} M_{\mathrm{AT}}=\frac{c}{4}\cdot R_{\mathrm{AB}}\cdot K_{A}\cdot \frac{\left(\frac{1}{c-v_{B12}}+\frac{1}{c-v_{A34}}-\frac{2}{c}\right)\cdot \left(T_{\mathrm{tof}2}+T_{\mathrm{tof}3}+T_{\mathrm{reA}}\right)+T_{\mathrm{reB}}\cdot \left(\frac{1}{c-v_{B56}}+\frac{1}{c-v_{A34}}-\frac{2}{c}\right)}{T_{\mathrm{tof}2}+T_{\mathrm{tof}3}+T_{\mathrm{reA}}+T_{\mathrm{reB}}}\\ +\frac{c}{4}\cdot R_{\mathrm{AB}}\cdot K_{B}\cdot \frac{\left(\frac{1}{c-v_{A34}}+\frac{1}{c-v_{B56}}-\frac{2}{c}\right)\cdot \left(T_{\mathrm{tof}1}+T_{\mathrm{tof}2}+T_{\mathrm{reB}}\right)+T_{\mathrm{reA}}\cdot \left(\frac{1}{c-v_{B12}}+\frac{1}{c-v_{A34}}-\frac{2}{c}\right)}{T_{\mathrm{tof}1}+T_{\mathrm{tof}2}+T_{\mathrm{reB}}+T_{\mathrm{reA}}}\\ +\frac{c}{4}v_{\mathrm{AB}35}\cdot \left(t_{5}^{}-t_{3}^{}\right)/\left(c-v_{B56}\right)\cdot \left[\frac{T_{\mathrm{reB}}\cdot K_{A}}{T_{\mathrm{tof}2}+T_{\mathrm{tof}3}+T_{\mathrm{reA}}+T_{\mathrm{reB}}}+\frac{\left(T_{\mathrm{tof}1}+T_{\mathrm{tof}2}+T_{\mathrm{reB}}\right)\cdot K_{B}}{T_{\mathrm{tof}1}+T_{\mathrm{tof}2}+T_{\mathrm{reB}}+T_{\mathrm{reA}}}\right]\\ -\frac{c}{4}v_{\mathrm{AB}13}\cdot \left(t_{3}^{}-t_{1}^{}\right)/\left(c-v_{B12}\right)\cdot \left[\frac{\left(T_{\mathrm{tof}2}+T_{\mathrm{tof}3}+T_{\mathrm{reA}}\right)\cdot K_{A}}{T_{\mathrm{tof}2}+T_{\mathrm{tof}3}+T_{\mathrm{reA}}+T_{\mathrm{reB}}}+\frac{T_{\mathrm{reA}}\cdot K_{B}}{T_{\mathrm{tof}1}+T_{\mathrm{tof}2}+T_{\mathrm{reB}}+T_{\mathrm{reA}}}\right]\\ =\frac{1}{4}\cdot R_{\mathrm{AB}}\cdot \left[\frac{\left(v_{A34}+v_{B12}\right)\cdot c-2v_{B12}\cdot v_{A34}}{\left(c-v_{B12}\right)\cdot \left(c-v_{A34}\right)}\right]\cdot \left[\frac{\left(T_{\mathrm{tof}2}+T_{\mathrm{tof}3}+T_{\mathrm{reA}}\right)\cdot K_{A}}{T_{\mathrm{tof}2}+T_{\mathrm{tof}3}+T_{\mathrm{reA}}+T_{\mathrm{reB}}}+\frac{T_{\mathrm{reA}}\cdot K_{B}}{T_{\mathrm{tof}1}+T_{\mathrm{tof}2}+T_{\mathrm{reB}}+T_{\mathrm{reA}}}\right]\\ +\frac{1}{4}\cdot R_{\mathrm{AB}}\cdot \left[\frac{\left(v_{B56}+v_{A34}\right)\cdot c-2v_{A34}\cdot v_{B56}}{\left(c-v_{A34}\right)\cdot \left(c-v_{B56}\right)}\right]\cdot \left[\frac{\left(T_{\mathrm{tof}1}+T_{\mathrm{tof}2}+T_{\mathrm{reB}}\right)\cdot K_{B}}{T_{\mathrm{tof}1}+T_{\mathrm{tof}2}+T_{\mathrm{reB}}+T_{\mathrm{reA}}}+\frac{T_{\mathrm{reB}}\cdot K_{A}}{T_{\mathrm{tof}2}+T_{\mathrm{tof}3}+T_{\mathrm{reA}}+T_{\mathrm{reB}}}\right]\\ +\frac{c}{4}v_{\mathrm{AB}35}\cdot \left(t_{5}^{}-t_{3}^{}\right)/\left(c-v_{B56}\right)\cdot \left[\frac{T_{\mathrm{reB}}\cdot K_{A}}{T_{\mathrm{tof}2}+T_{\mathrm{tof}3}+T_{\mathrm{reA}}+T_{\mathrm{reB}}}+\frac{\left(T_{\mathrm{tof}1}+T_{\mathrm{tof}2}+T_{\mathrm{reB}}\right)\cdot K_{B}}{T_{\mathrm{tof}1}+T_{\mathrm{tof}2}+T_{\mathrm{reB}}+T_{\mathrm{reA}}}\right]\\ -\frac{c}{4}v_{\mathrm{AB}13}\cdot \left(t_{3}^{}-t_{1}^{}\right)/\left(c-v_{B12}\right)\cdot \left[\frac{\left(T_{\mathrm{tof}2}+T_{\mathrm{tof}3}+T_{\mathrm{reA}}\right)\cdot K_{A}}{T_{\mathrm{tof}2}+T_{\mathrm{tof}3}+T_{\mathrm{reA}}+T_{\mathrm{reB}}}+\frac{T_{\mathrm{reA}}\cdot K_{B}}{T_{\mathrm{tof}1}+T_{\mathrm{tof}2}+T_{\mathrm{reB}}+T_{\mathrm{reA}}}\right]\\ \approx \frac{1}{2}\cdot R_{\mathrm{AB}}\cdot \frac{\left(K-N_{\mathrm{AB}}\right)\cdot \left(v_{A34}+v_{B12}\right)+N_{\mathrm{AB}}\cdot \left(v_{B56}+v_{A34}\right)}{K\cdot c}\\ +\frac{1}{2}\cdot \left(v_{\mathrm{AB}35}-v_{\mathrm{AB}13}\right)\cdot \frac{N_{\mathrm{AB}}\cdot \left(K-N_{\mathrm{AB}}\right)\cdot T_{s}^{}}{K} \end{array}$$

Similarly, the motion error for the time difference measurement can be simplified as:

(A12) $$\begin{array}{ll} M_{\Delta T} & =(T_{\mathrm{tof}2}-T_{\mathrm{tofAB}})\cdot K_{A}-\frac{1}{4}\cdot \left(T_{\mathrm{tof}1}+T_{\mathrm{tof}2}-2T_{\mathrm{tofAB}}\right)\cdot K_{A}-\frac{1}{4}\cdot \left(T_{\mathrm{tof}2}+T_{\mathrm{tof}3}-2T_{\mathrm{tofAB}}\right)\cdot K_{B}\\ & \;\;\;\;\;\;\;\;-\frac{1}{4}\cdot \left(T_{\mathrm{tof}3}-T_{\mathrm{tof}1}\right)\cdot \left(\frac{T_{\mathrm{reB}}\cdot K_{A}}{T_{\mathrm{tof}2}+T_{\mathrm{tof}3}+T_{\mathrm{reA}}+T_{\mathrm{reB}}}-\frac{T_{\mathrm{reA}}\cdot K_{B}}{T_{\mathrm{tof}1}+T_{\mathrm{tof}2}+T_{\mathrm{reB}}+T_{\mathrm{reA}}}\right)\\ & =\frac{1}{4}\cdot T_{\mathrm{tofAB}}\cdot \left(\frac{c}{c-v_{A34}}-1\right)\cdot K_{A}-\frac{T_{\mathrm{tofAB}}}{2}\cdot \frac{\left(K-N_{\mathrm{AB}}\right)\cdot \left(v_{A34}+v_{B12}\right)+N_{\mathrm{AB}}\cdot \left(v_{B56}+v_{A34}\right)}{K\cdot c}\\ & \;\;\;\;\;\;\;\;-\frac{v_{\mathrm{AB}35}-v_{\mathrm{AB}13}}{2\cdot c}\cdot \frac{N_{\mathrm{AB}}\cdot \left(K-N_{\mathrm{AB}}\right)\cdot T_{s}^{}}{K}\\ & \approx \frac{1}{4}\cdot T_{\mathrm{tofAB}}\cdot \frac{v_{A34}}{c-v_{A34}}-\frac{T_{\mathrm{tofAB}}}{2}\cdot \frac{\left(K-N_{\mathrm{AB}}\right)\cdot \left(v_{A34}+v_{B12}\right)+N_{\mathrm{AB}}\cdot \left(v_{B56}+v_{A34}\right)}{K\cdot c}\\ & \;\;\;\;\;\;\;\;-\frac{v_{\mathrm{AB}35}-v_{\mathrm{AB}13}}{2\cdot c}\cdot \frac{N_{\mathrm{AB}}\cdot \left(K-N_{\mathrm{AB}}\right)\cdot T_{s}^{}}{K} \end{array}$$

## Appendix C

The transmitter code-phase jitter noise affects $T_{\mathrm{rdA}}$, $T_{\mathrm{rdB}}$, $T_{\mathrm{reA}}$, and $T_{\mathrm{reB}}$, with the effect denoted as $\sigma_{\theta \mathrm{rdA}}$, $\sigma_{\theta \mathrm{rdB}}$, $\sigma_{\theta \mathrm{reA}}$, and $\sigma_{\theta \mathrm{reB}}$, respectively. The phase tracking error for $T_{\mathrm{rdA}}$, $T_{\mathrm{rdB}}$, $T_{\mathrm{reA}}$, and $T_{\mathrm{reB}}$ are denoted as $n_{\mathrm{rdA}}$, $n_{\mathrm{rdB}}$, $n_{\mathrm{reA}}$, and $n_{\mathrm{reB}}$, respectively, and they can be expressed as:

(A13) $$\begin{array}{l} n_{\mathrm{rdA}}^{}=\sqrt{\sigma_{\theta \mathrm{rdA}}^{2}+\sigma_{{}_{\mathrm{DLL}1}}^{2}}/\left(2\pi f_{\mathrm{PN}}\right)\\ n_{\mathrm{rdB}}^{}=\sqrt{\sigma_{\theta \mathrm{rdB}}^{2}+\sigma_{{}_{\mathrm{DLL}3}}^{2}}/\left(2\pi f_{\mathrm{PN}}\right)\\ n_{\mathrm{reA}}^{}=\sqrt{\sigma_{\theta \mathrm{reA}}^{2}+\sigma_{{}_{\mathrm{DLL}5}}^{2}}/\left(2\pi f_{PN}\right)\\ n_{\mathrm{reB}}^{}=\sqrt{\sigma_{\theta \mathrm{reB}}^{2}+(-\sigma_{{}_{\mathrm{DLL}3}}^{}{)}^{2}}/\left(2\pi f_{PN}\right) \end{array}$$

From [20] we know that

(A14) $$\begin{array}{l} R_{\mathrm{AT}}=\frac{c}{4}\cdot \left[\left(T_{\mathrm{tof}1}+T_{\mathrm{tof}2}+T_{\mathrm{reB}}\right)\cdot K_{A}+\left(T_{\mathrm{tof}2}+T_{\mathrm{tof}3}+T_{\mathrm{reA}}\right)\cdot K_{B}\right]\\ \;\;\;\;\;\;\;\;-\frac{c}{4}\cdot \left(T_{\mathrm{reB}}\cdot K_{B}+n_{\mathrm{reB}}\right)\cdot \frac{T_{\mathrm{AP}}\cdot K_{A}+\varepsilon_{\mathrm{rdA}}+n_{A}}{T_{\mathrm{BP}}\cdot K_{B}+\varepsilon_{\mathrm{rdB}}+n_{B}}+\frac{c}{4}\cdot \left(\varepsilon_{\mathrm{rdA}}+n_{\mathrm{rdA}}\right)\\ \;\;\;\;\;\;\;\;-\frac{c}{4}\cdot \left(T_{\mathrm{reA}}\cdot K_{A}+n_{\mathrm{reA}}\right)\cdot \frac{T_{\mathrm{BP}}\cdot K_{B}+\varepsilon_{\mathrm{rdB}}+n_{B}}{T_{\mathrm{AP}}\cdot K_{A}+\varepsilon_{\mathrm{rdA}}+n_{A}}+\frac{c}{4}\cdot \left(\varepsilon_{\mathrm{rdB}}+n_{\mathrm{rdB}}\right) \end{array}$$

where $\varepsilon_{\mathrm{rdA}}$ and $\varepsilon_{\mathrm{rdB}}$ are the residual errors of the hardware delay and ionospheric delay, respectively, which are very small and can be neglected. $T_{\mathrm{AP}}$, $T_{\mathrm{BP}}$, $n_{A}$, and $n_{B}$ can be expressed as:

(A15) $$\begin{array}{l} T_{\mathrm{AP}}=T_{\mathrm{tof}1}+T_{\mathrm{tof}2}+T_{\mathrm{reB}}+T_{\mathrm{reA}}\\ T_{\mathrm{BP}}=T_{\mathrm{tof}2}+T_{\mathrm{tof}3}+T_{\mathrm{reA}}+T_{\mathrm{reB}}\\ n_{A}=n_{\mathrm{rdA}}+n_{\mathrm{reA}}\\ n_{B}=n_{\mathrm{rdB}}+n_{\mathrm{reB}} \end{array}$$

Thus, $n_{R}$ can be derived as [20]:

(A16) $$\begin{array}{ll} n_{R}=\frac{c}{4}\cdot \left(n_{\mathrm{rdA}}+n_{\mathrm{rdB}}\right)-\frac{c}{4} & \cdot n_{\mathrm{reB}}\cdot \frac{T_{\mathrm{AP}}\cdot K_{A}+\varepsilon_{\mathrm{rdA}}+n_{\mathrm{rdA}}+n_{\mathrm{reA}}}{T_{\mathrm{BP}}\cdot K_{B}+\varepsilon_{\mathrm{rdB}}+n_{\mathrm{rdB}}+n_{\mathrm{reB}}}\\ & -\frac{c}{4}\cdot n_{\mathrm{reA}}\cdot \frac{T_{\mathrm{BP}}\cdot K_{B}+\varepsilon_{\mathrm{rdB}}+n_{\mathrm{rdB}}+n_{\mathrm{reB}}}{T_{\mathrm{AP}}\cdot K_{A}+\varepsilon_{\mathrm{rdA}}+n_{\mathrm{rdA}}+n_{\mathrm{reA}}}\\ & +\frac{c}{4}\cdot T_{\mathrm{reB}}\cdot K_{B}\cdot \left(\frac{T_{\mathrm{AP}}\cdot K_{A}+\varepsilon_{\mathrm{rdA}}}{T_{\mathrm{BP}}\cdot K_{B}+\varepsilon_{\mathrm{rdB}}}-\frac{T_{\mathrm{AP}}\cdot K_{A}+\varepsilon_{\mathrm{rdA}}+n_{\mathrm{rdA}}+n_{\mathrm{reA}}}{T_{\mathrm{BP}}\cdot K_{B}+\varepsilon_{\mathrm{rdB}}+n_{\mathrm{rdB}}+n_{\mathrm{reB}}}\right) \end{array}$$
